# Postmortem Changes in Skin Appendages of Domestic Dogs: A Preliminary Comparative Histological Study Between Body Regions, and Possible Forensic Implications

**DOI:** 10.3390/ani16193030

**Published:** 2026-09-26

**Authors:** Artemis Kalaitzi, Dimitrios Doukas, Georgios Ziagkas, Chara Spiliopoulou, Dimitrios Tontis

**Affiliations:** 1 Laboratory of Animal Pathology and Veterinary Forensics, Faculty of Veterinary Medicine, School of Health Sciences, University of Thessaly, 43100 Karditsa, Greece; ddoukas@uth.gr (D.D.); ziagkas@uth.gr (G.Z.); dtontis@uth.gr (D.T.); 2 Department of Forensic Medicine and Toxicology, School of Medicine, National and Kapodistrian University of Athens, 11527 Athens, Greece; chspiliop@med.uoa.gr

**Keywords:** dog, postmortem interval, PMI, veterinary forensics, histology, skin appendages, autolysis

## Abstract

This study describes postmortem histological changes in skin appendages of domestic dogs in the first seven days after death. The objective was to document the progression of autolytic change in the sweat glands, sebaceous glands, and hair follicles and to compare it between body regions and sides. Skin samples were collected bilaterally from the neck, thorax, abdomen, and thigh of nine dog carcasses (female, non-pedigree, short-haired, 5–10 years old, ~10 kg). The carcasses were kept at 10–12 °C in Karditsa, Greece, and sampled at 0, 4, 8, 12, 24, 48, 72, 96, 120, 144, and 168 h postmortem. Sections were stained with Hematoxylin and Eosin and Alcian Blue-PAS. The rate of change varied by structure, body region, and side. In this cohort, autolysis appeared earliest in the abdomen and on the dependent (left) side. The sweat glands and hair follicles reached the most advanced stage recorded by 48 h, whereas the sebaceous glands remained unchanged for 24 h, except in the dependent abdomen. The three structures changed over different periods, giving complementary timelines. These findings provide the first structured reference data on postmortem change in dog skin appendages, a baseline for early postmortem interval estimation and for recognizing antemortem lesions.

## Simple Summary

Histology is a useful tool in veterinary forensics, but few studies have examined how the microscopic structures of animal skin change after death. In this study, we documented changes in the skin appendages (sweat glands, sebaceous glands, and hair follicles) of nine dogs kept under cool conditions over the first seven days following death. The changes appeared in a consistent order within each structure, but the timing varied depending on the structure, body region, and side of the body. These observations provide reference data that may help veterinary pathologists estimate the earliest time since death and record how healthy skin appendages normally decompose, a first step towards distinguishing natural changes that occur after death from those caused while the animal was still alive.

## 1. Introduction

The skin is the largest organ of the dog’s body. It functions mainly as a protective barrier between the internal organs and the external environment [1]. The skin is also useful in forensic investigations because it can provide information on both the postmortem interval and on lesions related to the cause of death. It is structurally more robust than other soft tissues, resisting environmental and decompositional processes, and is easily accessible for sampling [2].

Determining the postmortem interval (PMI) is a core task for veterinary forensic pathologists. The postmortem interval, or “time since death,” is the amount of time that has passed since an animal died and its body was discovered [3]. The calculation of the postmortem period is usually an estimate unless the death was observed, and precision decreases as the postmortem interval extends [1,4].

PMI estimation in animals is more difficult and less commonly used than in humans due to species diversity and methodological differences [5]. In cases of intentional abuse (non-accidental injury) of companion animals, PMI estimation could help investigators narrow the “window of opportunity,” which would mean ruling out certain events and suspects and also establishing whether the animal suffered between the assault and its death [6]. In veterinary cases, it can also confirm the accuracy of insurance claims for an animal’s death, verify whether a hunted animal was killed during a legal hunting season, and establish the sequence of events for an animal and a person found dead simultaneously [7].

Τhe identification and accurate description of lesions at necropsy are important in veterinary forensic casework. The study of wound morphology, position, and whether it was inflicted before or after death provides information on the cause and manner of death and can indicate whether the wound was accidental or intentional [8,9]. Knowing the rate of postmortem changes also allows the veterinary pathologist to distinguish genuine antemortem injuries, sustained while the animal was alive, from those resulting from natural postmortem decomposition [10]. Misinterpreting natural changes can lead to incorrect conclusions about the circumstances of death and mislead the court. Accurate detection of these changes supports PMI estimation, which provides a forensic timeline in animal cruelty investigations [11].

The scientific literature on PMI estimation in domestic species is limited. Most research focuses on thermometric changes, the distribution of rigor mortis, K^+^ levels in cerebrospinal fluid, hypostasis, skin discoloration, and changes in internal organs, as evidenced by histopathological and immunohistochemical findings, including the progressive loss of B- and T-cell immunoreactivity [12]. Forensic entomology also estimates PMI in animals from the time of colonization (TOC), the point at which insects first reach the body [13]. Other approaches studied specifically in dogs include radiographic gas accumulation in the cardiac chambers [14] and skeletal muscle proteolysis [15].

Postmortem changes in animal skin are both macroscopic and microscopic. The macroscopic changes begin with livor mortis, particularly conspicuous in light-colored skin and skin mucous membranes, and proceed with gray-green discoloration, skin slippage, and hair loss as decomposition continues. Under extreme environmental conditions, the skin may also dehydrate and mummify in dry environments, or form adipocere when the body is immersed in water or kept in a wet, warm, anaerobic environment [4,16]. 

Although most research on postmortem changes has focused on gross anatomy, consistent changes also occur at the microscopic level. Because tissue autolyzes in a predictable, time-related manner, histology can help narrow the postmortem interval. Autolysis begins throughout the body at the same time, but cell types differ in how quickly they degrade, depending on their resistance to oxygen deprivation [17]. A sequence of such changes in the skin appendages over the postmortem period has been reported in human skin [18,19,20,21].

In animal species, however, histological data remain scarce. Postmortem histological changes in the skin of cattle have been shown to progress in a time-dependent manner related to the PMI [22]. Comparable reference patterns for dog skin are lacking. The postmortem changes in the dog skin appendages have not been documented, so it is not known whether they follow the same time-dependent pattern seen in other species or differ by region and side. Without this baseline, antemortem lesions cannot be separated from normal autolysis. This is a notable gap, since dogs are among the species most often encountered in veterinary forensic investigations, through their close association with humans and their frequent involvement in abuse cases.

Because tissue autolyzes in a time-dependent manner, and the appendages, skin thickness, and local tissue environment all vary across the body, we hypothesized that the dog skin appendages autolyze in a consistent order, at a rate that differs by appendage, body region, and side. The focus of this preliminary study is to identify the time-related postmortem histologic changes in dog skin appendages (hair follicles, sweat glands, and sebaceous glands). This study documents the rate at which these changes occur, establishes baseline information for veterinary forensics, and evaluates the usefulness of examining histologic sections of skin for estimation of postmortem interval (PMI). Unlike previous studies, this one evaluated several locations on each dog’s body to determine whether postmortem changes occur similarly or whether the rate of autolysis differs by site and/or side.

## 2. Materials and Methods

### 2.1. Animal Collection

The present study was carried out at the Necropsy Hall of the Laboratory of Animal Pathology and Veterinary Forensics, University of Thessaly, Greece, between December 2025 and February 2026.

Nine female non-pedigree short-haired domestic dogs (*Canis lupus familiaris*) were selected for use in this study. As the dogs were unowned and no birth records were available, their age was estimated from dental wear at 5–10 years. Their average body condition score (BCS) rating was 5/9, and their average body weight was about 10 kg. Individual characteristics of each animal are given in Table 1. The first animals that were available and not excluded according to the study criteria happened to be female, and only females were used thereafter to ensure cohort homogeneity regarding sex.

The cadavers were donated to the Laboratory of Animal Pathology and Veterinary Forensics by veterinary clinics in the Karditsa area. All dogs had been euthanized on the attending veterinarian’s recommendation for incurable non-infectious conditions. The traumatic conditions (spinal cord, brain, and mandibular injury) were localized to the skeleton and nervous system, remote from the trunk skin sampled. The referring clinics reported the reason for euthanasia and confirmed that no animal had clinical evidence of sepsis or systemic infection that might have accelerated postmortem decomposition. No detailed prior clinical records were available, however, so any treatment the animals may have received before presentation is unknown.

The following exclusion criteria were applied: (1) carcasses whose precise moment of death was unknown; (2) carcasses with generalized or local skin changes or severe skin conditions; (3) carcasses with burns; (4) carcasses recovered from water; (5) severely dehydrated carcasses; and (6) animals whose recorded cause of euthanasia was a systemic disease.

The participating clinics were located close to the laboratory (approximately 2 km) and notified the team before each euthanasia so that a member of the research team was present and the time of death was recorded. The attending veterinarian confirmed death by auscultation, noting the absence of a heartbeat. The same procedure was followed for all nine animals. After confirmation of death, the cadavers were transported to the Necropsy Hall by car at ambient winter temperature, comparable to the storage conditions, and the time-zero (baseline) samples were taken there in less than 10 min from death; this short interval was the same for all nine animals. Skin samples were fixed in formalin immediately after collection. The appendages were at stage 0 at this time.

The carcasses were positioned in open-topped plastic trays in left lateral recumbency. Left lateral recumbency was chosen because it is the standard positioning for small-animal necropsy, so that body position was identical across the cohort and the dependent (left) and non-dependent (right) sides were defined consistently in every animal. Recumbency was not alternated or randomized in this preliminary study. 

The trays were placed in a well-ventilated 5 × 5 m area adjacent to the necropsy hall. The room itself was enclosed and insects could not enter, so no insect activity, larvae, or eggs were observed on any carcass at any interval, consistent with the winter season and the low storage temperature.

Room temperature and humidity were recorded daily. Ambient temperature remained within a narrow range of 10–12 °C throughout the study, while relative humidity remained between 45 and 65%. These conditions were consistent across the study period and the same for all nine carcasses.

### 2.2. Sample Selection and Processing

The body sites chosen for sampling included several anatomical regions and both sides of the body, rather than a single location, so that regional and position-related differences in autolysis could be assessed. Full-thickness skin samples were obtained using an 8 mm biopsy punch (Kruuse, Langeskov, Denmark) from the neck, thorax, thigh, and abdomen, on both the left (dependent) and right (non-dependent) sides of the body. This resulted in eight sampling sites at each time point (Figure 1), which were sampled at 0, 4, 8, 12, 24, 48, 72, 96, 120, 144, and 168 h postmortem (8 sites × 11 time points = 88 samples per animal; 88 × 9 animals = 792 samples in total). 

Each sample was taken from intact skin at least 1 cm from previous biopsy sites, to prevent local skin damage caused by repeated sampling from being confused with postmortem changes. The punch defects were not sutured and remained open for the rest of the study. None of the sampling sites had visible lesions at the start of the study. Therefore, any microscopic changes observed could be attributed to postmortem autolysis rather than to any pre-existing dermatological condition.

The samples were carefully processed to avoid traumatic artifacts during collection. They were placed in a plastic container and fixed in 10% neutral-buffered formalin prepared from 37% formaldehyde (BioGnost, Zagreb, Croatia). After fixation, the samples were routinely embedded in paraffin (Leica Biosystems, Richmond, IL, USA) and sectioned at 5 μm with disposable microtome blades (Micros, St. Veit an der Glan, Austria). Sections were mounted on adhesive microscope slides (Süsse Labortechnik, Gudensberg, Germany) to reduce detachment of the autolyzed tissue. They were stained with H&E, using Harris’s hematoxylin (Labbox Labware, Premià de Dalt, Spain) and eosin Y (BioGnost), and with an Alcian Blue-PAS kit (BioGnost). Sections were examined under a Nikon Eclipse Si microscope (Nikon Corporation, Tokyo, Japan) equipped with a Euromex CMEX-3PRO digital camera (Euromex Microscopen B.V., Arnhem, The Netherlands), and images were captured using ImageFocus Alpha software (version 1.3.7; Euromex).

Alcian Blue-PAS staining was used in addition to H&E to examine the secretory cells of the sweat glands and the basement membrane. The combined stain allows better identification of the secretory cells and their postmortem loss of staining to be followed, as described in previous studies of the sweat glands [18,19]. Periodic acid-Schiff also stains the basement membrane because of its high glycoprotein content, allowing its PAS positivity to be recorded across postmortem intervals. The secretory cells were classified as positive when their cytoplasm showed the characteristic magenta PAS reaction and as negative when this was absent. The basement membrane was classified as positive when it showed the same magenta PAS reaction at the epithelial base. The assessment was qualitative and binary, with no intermediate grades.

### 2.3. Histomorphological Staging

For the purposes of the present study, histological staging was based on the skin appendages. The surrounding epidermis and dermis were not assessed because their postmortem changes were difficult to distinguish from processing artifacts. The appendages showed a clear, repeatable sequence of change across intervals and were more suitable for grading. The epidermis and dermis remain to be addressed in future work.

Histomorphological staging and histochemical (Alcian Blue-PAS) assessment were regarded as separate systems of evaluation: the first evaluates the structural autolytic alterations observable in hematoxylin and eosin-stained sections, while the second assesses the PAS staining of the sweat gland secretory cells. The results of Alcian Blue-PAS staining were therefore documented separately and not incorporated into the morphologic staging. Alcian Blue-PAS staining was performed on all eight sampling sites at all eleven time points.

Autolytic change in the skin appendages was assessed with a qualitative staging system based on predetermined morphologic criteria. Separate criteria were used for the sweat glands, sebaceous glands, and hair follicles, because the three structures differ in organization. Each section corresponded to one location in one animal at a given postmortem interval and was assessed on its own. The staging criteria for each structure were based on published histological indicators of postmortem autolysis in skin appendages [18,19,20,21,22], from which the most distinctive and consistently identifiable features were selected. The assignment of each stage was agreed upon by consensus between two of the authors; since this study was designed to be descriptive, no inferential statistical analyses were employed. Each reported time corresponds to the earliest sampling interval at which the defining features of a stage were consistently identifiable in the sections, rather than a time reached uniformly across all material. It therefore indicates a minimum interval since death—that is, that at least this interval had elapsed—rather than an exact time or a population-wide threshold. The sequence of change followed the same order in all specimens. 

The staging relied on cytoplasmic vacuolization and nuclear changes, common to all three structures, together with additional features specific to each, as summarized in Table 2 and Table 3, and Table 4 for the sweat glands, sebaceous glands, and hair follicles, respectively. These features were chosen as clear, easily recognizable, and consistent markers of autolytic change, distinct from processing artifacts. In the tables showing the distribution of stages (Section 3), each cell shows a single stage assigned by consensus for that site and time point, not an individual animal’s stage or an average of the nine. A stage was recorded when its defining features were identifiable in the sections at that interval; this reflects the earliest point at which the stage could be recognized in the material, not its presence in every animal.

Only the secretory tubules of the epitrichial (apocrine) sweat glands were assessed, as the ducts were not consistently present in the sections.

In the sebaceous glands, two stages were used rather than three. Once cytoplasmic vacuolization appeared, its extent did not permit a consistent separation of early from advanced stages, so a single stage of change was recorded. This change was identified by coarse, sharply outlined cytoplasmic vacuoles, distinct from the fine, foamy cytoplasm of the normal sebocyte, together with nuclear pyknosis or karyolysis in peripheral sebocytes. Peripheral sebocytes were assessed because central sebocytes typically disintegrate during holocrine secretion.

Vacuolization and nuclear changes in hair follicles were assessed at the level of the isthmus. The isthmus is part of the permanent component of the follicle and is found in every stage of the hair growth cycle (telogen, anagen, and catagen). In contrast, the bulb degenerates during catagen and is missing during the telogen phase [23]. Staging was therefore based mainly on the outer root sheath at the isthmus, but did not assess whether an individual follicle was undergoing a particular phase of the hair growth cycle. Vacuolization caused by autolysis was distinguishable from the normal morphology of keratinocytes in the outer root sheath by comparison with sections obtained at 0 h after death, where the keratinocytes in the outer root sheath have compact eosinophilic cytoplasm and normal nuclei. Vacuoles were recorded if they appeared as distinct, rounded areas of clarity within the cytoplasm, often displacing the nucleus. Criteria related to the inner root sheath or bulb were used only if these components existed in the follicle being scored. Primary and secondary follicles were not differentiated.

## 3. Results

Figure 2 shows the timing of change at each site for the three structures.

### 3.1. Sweat Glands

At 0 h, the sweat glands in all regions displayed normal architecture with intact epithelium (Stage 0, Table 5, Figure 3A). Changes in Stage 1 were observed at 4 h across all locations and on both sides, and remained the same at 8 h. At this stage, the epithelial cells showed cytoplasmic vacuolization and sporadic nuclear pyknosis, with widening of the interstitial gaps between cells (Figure 3B). 

Stage 2 (Figure 3C) first developed in the abdomen, at 12 h on the left and 24 h on the right. Stage 2 was defined by loss of the normal glandular architecture, with detachment and collapse of the secretory epithelium into the lumen and nuclear pyknosis or karyolysis in most epithelial cells. Where cells remained attached to the basement membrane, they showed cytoplasmic vacuolization, and the interstitial gaps between them persisted. The thorax was next at 24 h and 48 h, and the neck and thigh reached Stage 2 bilaterally. No area advanced beyond Stage 2 up to 168 h. The dependent (left) side always preceded or coincided with the non-dependent side. This asymmetry was confined to the abdomen and thorax; the neck and thigh proceeded symmetrically.

Alcian Blue-PAS staining was used to assess the PAS positivity of the sweat gland secretory cells. The secretory cells were positive at 0 h (Figure 4A,B), 4 h (Figure 4C,D), and 8 h, and had lost their positivity by 12 h (Figure 4E). The basement membrane, by contrast, remained positive at every interval examined, up to 168 h. The same pattern was found in every region, on both sides of the body. Table 6 summarizes the staining by PMI.

### 3.2. Sebaceous Glands

The sebaceous glands remained normal (Stage 0, Table 7, Figure 5A) in all regions except the dependent(left) abdomen throughout the first 24 h, with the typical foamy cytoplasm of the sebocytes. Stage 1 (Figure 5B) showed coarse, sharply outlined cytoplasmic vacuoles, distinct from the normal foamy cytoplasm, with nuclear pyknosis or karyolysis in the peripheral sebocytes. It appeared earliest in the dependent (left) abdomen at 24 h, followed by the dependent (left) thorax and the non-dependent (right) abdomen at 48 h, and in the neck, the non-dependent (right) thorax, and both thighs at 72 h. These were the last of the three structures to show autolytic change, and the change followed the same lateral pattern seen in the other structures, appearing on the dependent (left) side of the trunk before the non-dependent (right) side.

### 3.3. Hair Follicles

At 0 h, the hair follicles showed normal architecture in all regions. The outer root sheath cells at the isthmus had compact eosinophilic cytoplasm and normal nuclei (Stage 0, Table 8, Figure 6A,B). In Stage 1, vacuoles and nuclear pyknosis appeared in sporadic outer root sheath cells at the isthmus, while the bulb cells kept their normal form. This first became visible in the dependent (left) abdomen at 4 h, while all other regions remained at Stage 0. Stage 1 extended to the remaining regions between 12 and 24 h: the left thorax at 12 h, and the neck, right thorax, right abdomen, and both thighs at 24 h (Figure 6C).

Progression to Stage 2 was rapid. In Stage 2, most of the outer root sheath cells at the isthmus showed vacuolization and nuclear pyknosis or karyolysis, with sporadic pyknosis in the hair bulb cells where present. The left abdomen again changed first, reaching Stage 2 at 24 h, with all remaining regions following at 48 h. No further progression was seen up to 168 h (Figure 6D,E). As with the other appendages, the dependent (left) abdomen showed the first change at each stage. The left thorax also preceded the right, whereas the neck and thighs progressed symmetrically; this lateral asymmetry was confined to two of the four regions (abdomen and thorax).

## 4. Discussion

Estimation of the postmortem interval is a common challenge in veterinary forensics. Yet few histological studies of the skin for this purpose have been conducted, and those that exist are almost entirely in humans. There is no equivalent study examining dog skin appendages that provides baseline data. The present study addresses this gap by providing a preliminary, descriptive characterization of the sequence of postmortem histological changes in the appendage structures of dog skin over 168 h under stable low-temperature conditions (10–12 °C).

As our observations indicate, the three skin appendages autolyzed at different rates. The sweat glands and hair follicles reached Stage 2 first at 48 h in most areas, but earlier on the dependent (left) side, with the abdomen reaching this stage within 12–24 h. The sebaceous glands were more resistant, showing autolytic change in the dependent (left) abdomen at 24 h, in the dependent (left) thorax and the non-dependent (right) abdomen at 48 h, and in the remaining regions at 72 h. The dependent (left) abdomen was generally the first site to progress. There was a lateral asymmetry, with changes occurring earlier on the dependent (left) side than on the non-dependent (right) side throughout the trunk, i.e., the abdomen and thorax.

In dogs, the most directly comparable study [24] did not find uniform changes in the skin after death. Instead, they examined the dogs’ internal organs and provided detailed information about the timing of their postmortem changes. However, neither the structures assessed nor the criteria used were specified for the skin, so it is not possible to say what was examined or how. It was also unlikely that the design would have identified the changes of interest here: skin samples were taken from an unspecified site on carcasses lying on their right sides. Dependent and non-dependent sites were not compared. Two animals were sampled at a time; sampling did not begin until 24 h after death, and the intervals between samples were too long to detect early changes. A negative result under these conditions does not exclude early, regional, or position-dependent change in the skin appendages. The current study found a clear temporal sequence by examining the sweat glands, sebaceous glands, and hair follicles separately, taking samples in the hours following death, and comparing dependent and non-dependent sites between several body regions.

The present study documents the progression of postmortem changes in dog skin, comparing dependent and non-dependent body-side regions. The histological criteria of the staging system, as described in human and animal postmortem skin studies, were identifiable in dog skin. The exact timing of postmortem changes is variable between studies, primarily due to ambient temperature and different species, but the order of autolysis of the appendage structures is broadly agreed upon in the literature [18,19,20,21,22], and our findings are consistent with this.

The sweat glands were one of the earliest structures to show autolytic changes, with cytoplasmic vacuolization present in all regions by 4 h. This is also one of the first cellular changes detected in human postmortem skin. The early susceptibility of these glands has been a recurring observation, even when low temperatures delay it to around 24 h [18,19,21]. Progression to Stage 2 was regional and side-dependent. The dependent (left) abdomen reached it by 12 h, the dependent (left) thorax and the non-dependent (right) abdomen by 24 h, and the remaining regions, including the neck and thighs, by 48 h. Considering this variation, our findings are compatible with those studies. Early autolysis of the sweat glands is a common feature of cutaneous autolysis and is not species-specific.

The progressive loss of PAS positivity in the secretory cells of the sweat glands accompanies the autolysis and complements the morphological changes seen on H&E. In our study, the secretory cells remained positive at 8 h and became PAS-negative by 12 h across all eight sites, irrespective of body position. The histochemical changes are therefore uniform, while the morphological ones are not. The staining disappeared in all regions by 12 h, whereas Stage 2 was reached only in the dependent (left) abdomen at that time and in the remaining regions at 24–48 h. The histomorphological autolytic changes, therefore, appeared later than the loss of staining, except in the dependent (left) abdomen, where the two coincided. This difference probably reflects a different underlying mechanism. Local factors such as hypostasis or proximity to the gastrointestinal tract influence the structural breakdown of the cells. The early disappearance of intracellular glycoprotein staining seems less sensitive to these factors, perhaps because it results from a hydrolytic process that occurs within the tissue soon after death. The staining is also scored only as present or absent and therefore cannot show the graded regional differences detected by the morphological staging, as explained below.

The timing of loss of PAS positivity in our study was later than in the study by Cingolani et al. [20], which reported that the secretory cells of human eccrine sweat glands lose PAS positivity within the first 6 h after death, and considerably later than in studies conducted under warmer conditions. Bardale et al. [19] showed that secretory cells lost positivity at 2–3 h postmortem, and a similar 2–3 h period was observed in cattle skin by Surendhar et al. [22]. The earlier loss most likely reflects the high ambient temperatures in those studies (23–37 °C), well above the 10–12 °C observed in the present study. These results contradict Babu et al. [18], who observed negative PAS staining of the secretory cells regardless of the postmortem interval in human skin samples.

Our basement membrane findings, PAS-positive up to 168 h, are in agreement with Cingolani et al. [20] and Babu et al. [18], who reported a similar contrast between PAS-negative secretory cells and a persistently PAS-positive basement membrane. However, their material was followed up to 12 h and 24 h, respectively. Surendhar et al. [22] found a similar pattern in cattle skin, where the basement membrane remained positive even after three days of death. Alcian Blue-PAS should be considered a binary, rather than a graded, marker: it distinguishes between intervals of less than 12 h and those longer than 12 h but provides no further temporal information. Within these limits, it is only an adjunct to the morphologic staging.

The three structures differed in the timing of autolytic changes. The sebaceous glands were the last of the three structures to change, remaining normal for the first 24 h at all sites except the dependent (left) abdomen, while the sweat glands had begun by 4 h. This resistance is a constant feature of the literature. The sebaceous glands were not the first appendage to change in any of the reported studies: in human skin, they degenerated after the sweat glands at both low and high ambient temperature [19,20,21], and in cattle, they were among the last structures to disintegrate, although there they autolyzed in parallel with the sweat glands rather than outlasting them [22]. What differs here is the size of the interval between the sebaceous glands and the other two structures. The sebocytes have a holocrine, lipid-rich cytoplasm, which offers a poor aqueous substrate for the hydrolytic enzymes responsible for early autolysis and may explain their resistance. The same lateral asymmetry appeared in the sebaceous glands, with the dependent (left) abdomen changing first. That even the most resistant of the three structures followed this pattern further suggests that body position may influence the rate of autolysis.

The hair follicles autolyzed on a similar timescale to the sweat glands and earlier than the sebaceous glands, and showed a marked regional difference, with onset ranging from 4 h in the abdomen to 24 h in the other regions. In the dependent (left) abdomen, follicular vacuolization at the isthmus was already observed 4 h after death, and Stage 2 was reached at 24 h. The dependent (left) thorax showed vacuolization by 12 h. In the remaining regions, vacuolization appeared around 24 h, and Stage 2 was reached by 48 h.

In human skin, hair follicles autolyze well after the sweat glands: Bardale et al. [19] found both to remain normal for 18 h, with autolysis beginning in both thereafter; Babu et al. [18] reported that both showed only minimal changes; and Wang et al. [21] described little change in the follicles before 72 h and in the sebaceous glands before 84 h, against 24 h for the sweat glands. In cattle, the reverse is seen, with vacuolization of the hair follicles at 8 h and of the sweat and sebaceous glands only at 24 h [22]. The present results are consistent with the latter. This difference is probably due to the portion of the hair follicle examined. Wang et al. [21] ascribed the synchrony between the follicles and the epidermis to the follicular infundibulum, whose epithelium is the same as that of the epidermis, and their material showed little change in the epidermis before 84 h. Thus, staging of the infundibulum records epidermal rather than follicular change. Staging here was limited to the isthmus and bulb and did not include the infundibulum.

The autolytic changes were not uniform throughout the body; there was a regional and lateral gradient. In this cohort, the abdomen showed the earliest autolytic change in all three structures, whereas the neck and thighs changed latest. A likely contributing factor is the proximity of the abdomen to the gastrointestinal tract. The bacteria in the gastro-intestinal tract continue to grow and produce gas after animals die [25]. The skin on the dorsal side of the neck, trunk, and tail is thicker than the skin on the abdomen, flanks, or medial sides of the limbs [26]. The gases, hydrolytic enzymes, and bacteria released there may accelerate autolysis in the overlying tissue. The dependent side is also subject to hypostasis and to sustained compression against the supporting surface. The pooled blood and fluid provide a richer substrate for autolytic and bacterial activity and a hypoxic microenvironment, both of which may favor earlier autolysis.

The same left-sided predominance appeared in the thorax, even though the heart and lungs autolyze slowly and lack the rapid, bacterially driven autolysis of the gastrointestinal tract. Because all carcasses lay on the left, dependency remained confounded by anatomical side, and we cannot fully separate a gravitational from an anatomical cause: in every carcass, the dependent side was also the anatomically left side, where the heart sits and the asymmetrical canine lungs lie. Hypostasis would produce earlier change on the dependent surface whatever the underlying organ, whereas an anatomical cause would follow the distribution of the viscera. That the slowly autolyzing thorax still changed first on the left points to body position rather than to the viscera. This remains a hypothesis, and a definitive separation would require some carcasses to be placed in right lateral recumbency, which was not done here.

Overall, the asymmetry was limited to the trunk. On either side, the neck and thighs changed at the same rate, probably a function of where the blood pools after death. The trunk contains the major vessels and blood-rich organs, so more blood pools on the lower side than in the neck or thighs.

These findings have several practical applications in forensic casework. Since the rate of autolysis depends on many circumstances, a given stage is best taken as proof that at least a specific period has passed, rather than the actual moment of death. Because the three skin appendages changed at different times within the first three days, staging them together appeared to narrow the interval more than any single structure. As the dependent side autolyzed earlier than the non-dependent side, the carcass’s recumbency should be recorded. Because the dependent side tended to change earlier, the difference between the two sides may also indicate the position in which a carcass has been lying, or suggest that it has been moved, adding information beyond the estimation of the postmortem interval. Another possible application is distinguishing between postmortem autolysis and antemortem pathology, a recognized goal of forensic histopathology, as autolytic change can otherwise resemble or mask true lesions [22,27]. A reliable description of the normal postmortem degeneration of these structures, as provided here for dogs, is a prerequisite for recognizing the histological appearance when it deviates from the expected autolytic pattern. The staining techniques used are also widely available, inexpensive, and frequently employed in diagnostic pathology. Consequently, the approach could be integrated into standard practice without specialized equipment.

Immunohistochemistry was not used in this study, primarily because it requires validated antibodies and specialized protocols that are less readily available than routine histological and histochemical staining. Immunohistochemical markers have nonetheless been used to estimate the postmortem interval from skin: De-Giorgio et al. [2] investigated whether the immunohistochemical detection of HMGB1 and its related proteins, Beclin1 and RAGE, could serve as a tool for estimating the postmortem interval, and reported a time-dependent shift in the HMGB1 signal from the nucleus to the cytoplasm with its progressive loss as the postmortem interval increased. For the appendages examined here, Cingolani et al. [20] reported that immunohistochemical markers added no time-dependent information beyond hematoxylin and eosin. Immunohistochemical markers could complement our findings in future work.

This study has several limitations. It was based on nine carcasses that were homogeneous in sex, breed, coat type, body condition, and age, all being non-pedigree, short-haired females aged 5–10 years. Such homogeneity may limit the applicability of the results to the general dog population, and confirming these findings will require larger, more varied cohorts. The carcasses were also kept at a narrow, low temperature range (10–12 °C). Because autolysis is strongly temperature-dependent, these timings apply to cool conditions only and cannot be extrapolated to warmer climates, where the changes would be expected to appear earlier. The animals had been euthanized for a range of incurable non-infectious conditions rather than a single cause, so the influence of different causes of death on the rate of autolysis could not be formally assessed. All dogs were euthanized with the same agent, pentobarbital sodium. As the same agent was used in every animal, it is unlikely to contribute to the differences observed between regions, sides, or appendages. Pentobarbital acts on the central nervous system and is not directly cytotoxic to the skin. An effect of the drug on the tissue was not specifically assessed and cannot be excluded, but is unlikely to have influenced the autolytic sequence. Because the dogs were unowned, their prior clinical history and any treatment received before euthanasia were unknown and a possible effect of previous medication on postmortem change therefore cannot be excluded. The biopsy defects also were left open, and whether they altered the microenvironment of adjacent skin was not assessed; each sample was, however, taken from intact skin separated from earlier defects.

An additional limitation relates to the histomorphological assessment. The staging was qualitative, which means it carries a degree of subjectivity, as histological assessment of the postmortem interval is known to depend on the examiner’s judgment [18,21]. To reduce this, each stage was assigned by consensus of two experienced observers according to predefined criteria; interobserver agreement was therefore not quantified. In addition, per-animal stage distributions and morphometric measurements were not recorded and could not be derived retrospectively. All carcasses were also examined in left lateral recumbency, so dependency was confounded by anatomical side; as discussed above, the lateral differences are best regarded as a hypothesis to be confirmed under opposite or randomized recumbencies. The reported times represent a minimum interval since death and should be interpreted with caution in forensic casework. The staging has not yet been validated on carcasses whose postmortem interval is unknown to the observer, so prospective blinded testing of this kind is a necessary next step. Independent blinded scoring, quantitative morphometry on larger cohorts, and testing under randomized recumbency are all priorities for future work.

## 5. Conclusions

This study systematically documents the postmortem autolysis of dogs’ skin appendages and shows that this process is neither uniform nor synchronous. The rate varied according to the skin structure studied, the region of the body, and the carcass’s position after death.

The study focused on skin appendages: the sweat glands, sebaceous glands, and hair follicles. The three structures autolyzed at different rates. Sebaceous glands changed later than sweat glands and hair follicles. Because the three structures autolyzed at different rates, examining them together may refine the estimation of time since death compared with any one alone, although this combined approach was not validated here. Autolysis also appeared earlier in the abdomen than in the neck and thighs, and, in this cohort, earlier on the dependent (left) side of the trunk than on the non-dependent (right) side, a difference likely related to body position, though this needs confirmation under randomized recumbency.

These results may help estimate the postmortem interval in dogs. The structure examined, the region sampled, and the body position all affect the histological estimate of time since death, which reflects a minimum interval rather than the exact time of death. No single method can establish the postmortem interval on its own, and skin histology provides a more reliable estimate only when combined with other methods. The reference patterns also help separate postmortem autolysis from antemortem pathology.

Our findings are preliminary and require validation in larger and more heterogeneous cohorts including both sexes, different breeds and coat types, a range of ages and body conditions, alternating recumbency on both sides, and multiple ambient temperature ranges. Even so, they provide a reference for future work and a practical basis for using skin histology to estimate the early postmortem interval in veterinary forensic practice.

## Figures and Tables

**Figure 1:**
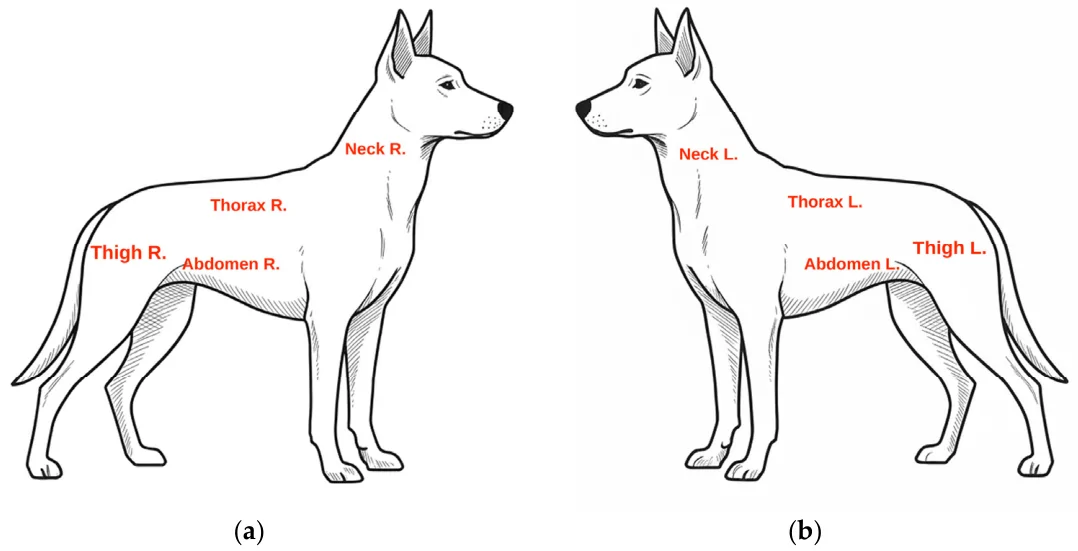
Anatomical location of the eight sampling sites. (**a**) Sampling sites on the right (R) lateral aspect of the dogs. (**b**) Sampling sites on the left (L) lateral aspect of the dogs.

**Figure 2:**
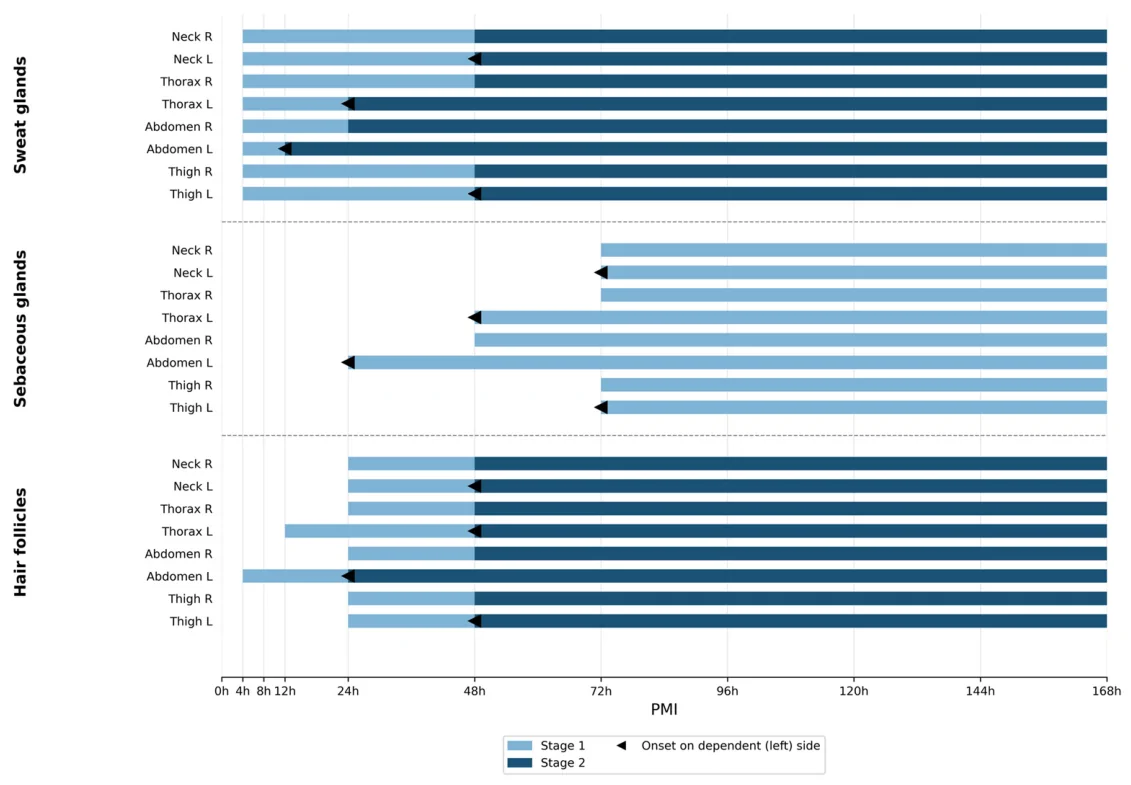
Timing of postmortem autolytic change in the three skin appendages across the eight sampling sites, from 0 to 168 h postmortem. Each horizontal bar is one site. Sites are grouped by structure and, within each structure, arranged in four regions (neck, thorax, abdomen, thigh), each with its right (R) and left (L) side shown separately. Light shading marks Stage 1 and dark shading marks Stage 2. The arrowhead marks the onset of the most advanced change on the dependent (left) side.

**Figure 3:**
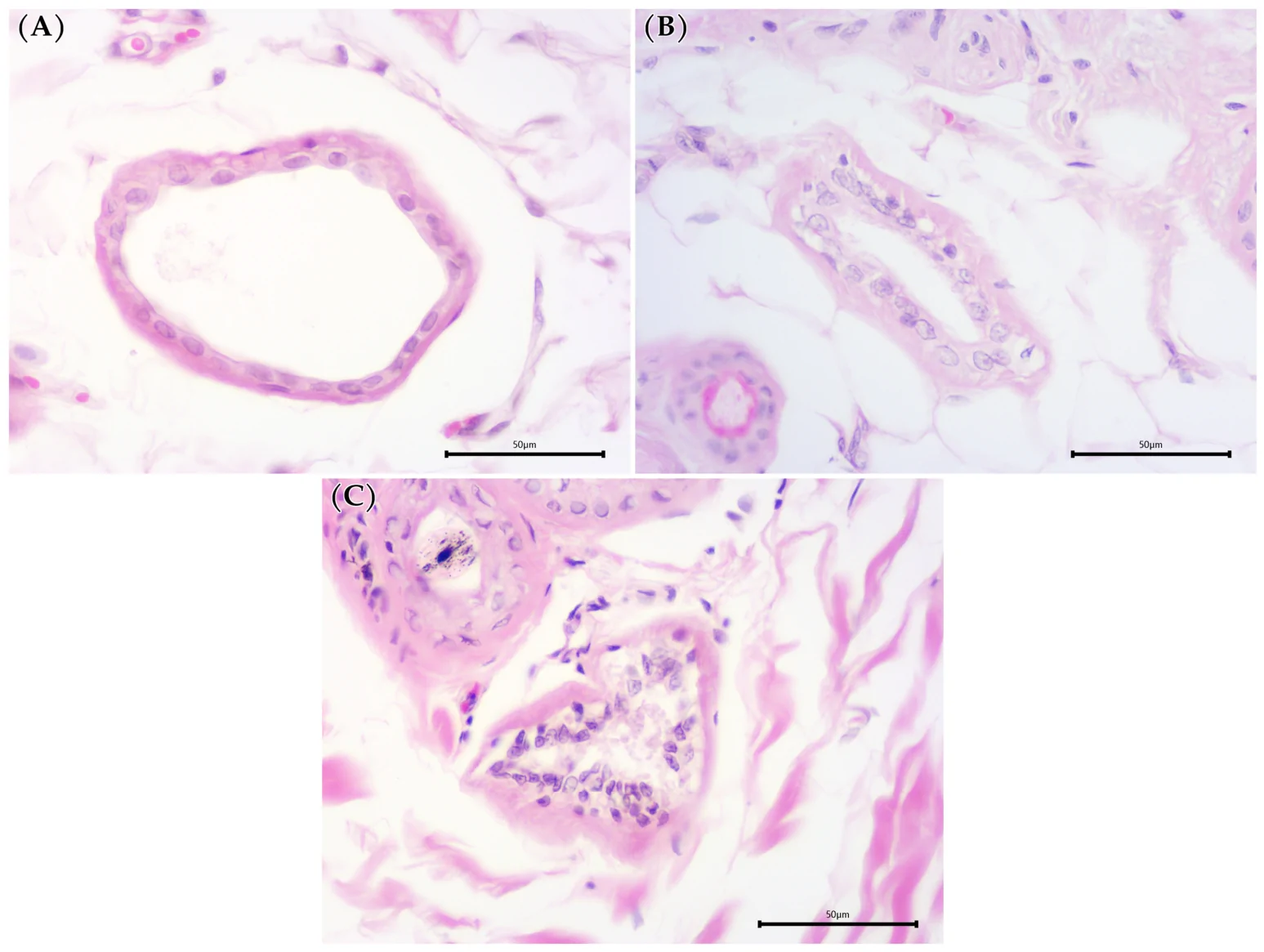
Postmortem autolytic stages of the sweat glands (H&E). (**A**) Normal architecture with intact secretory epithelium at 0 h (Stage 0), neck left, scale bar = 50 µm; (**B**) cytoplasmic vacuolization, sporadic nuclear pyknosis and widening of the interstitial gaps between cells at 12 h (Stage 1), thigh right, scale bar = 50 µm; (**C**) nuclear pyknosis in most epithelial cells and loss of glandular architecture, with detachment and collapse of the secretory epithelium into the lumen at 72 h (Stage 2), abdomen right, scale bar = 50 µm.

**Figure 4:**
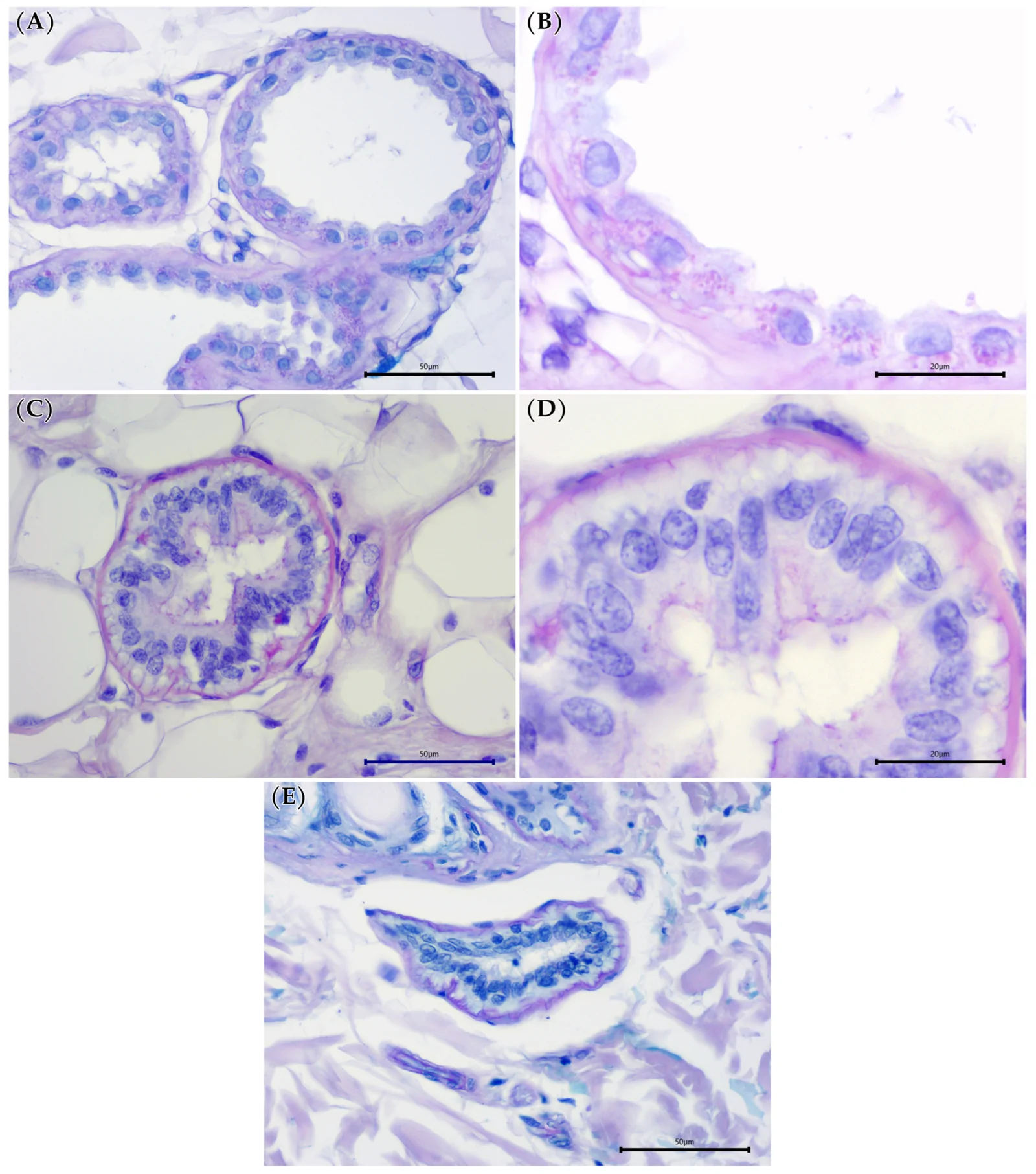
Postmortem changes in sweat gland glycoprotein content assessed by Alcian Blue-PAS staining. (**A**) Secretory cells and basement membrane PAS-positive at 0 h, abdomen left, scale bar = 50 µm; (**B**) same at higher magnification, scale bar = 20 µm; (**C**) secretory cells still PAS-positive at 4 h, abdomen right, scale bar = 50 µm; (**D**) same at higher magnification, scale bar = 20 µm; (**E**) loss of PAS positivity in the secretory cells with retained positivity of the basement membrane at 12 h, thorax left, scale bar = 50 µm.

**Figure 5:**
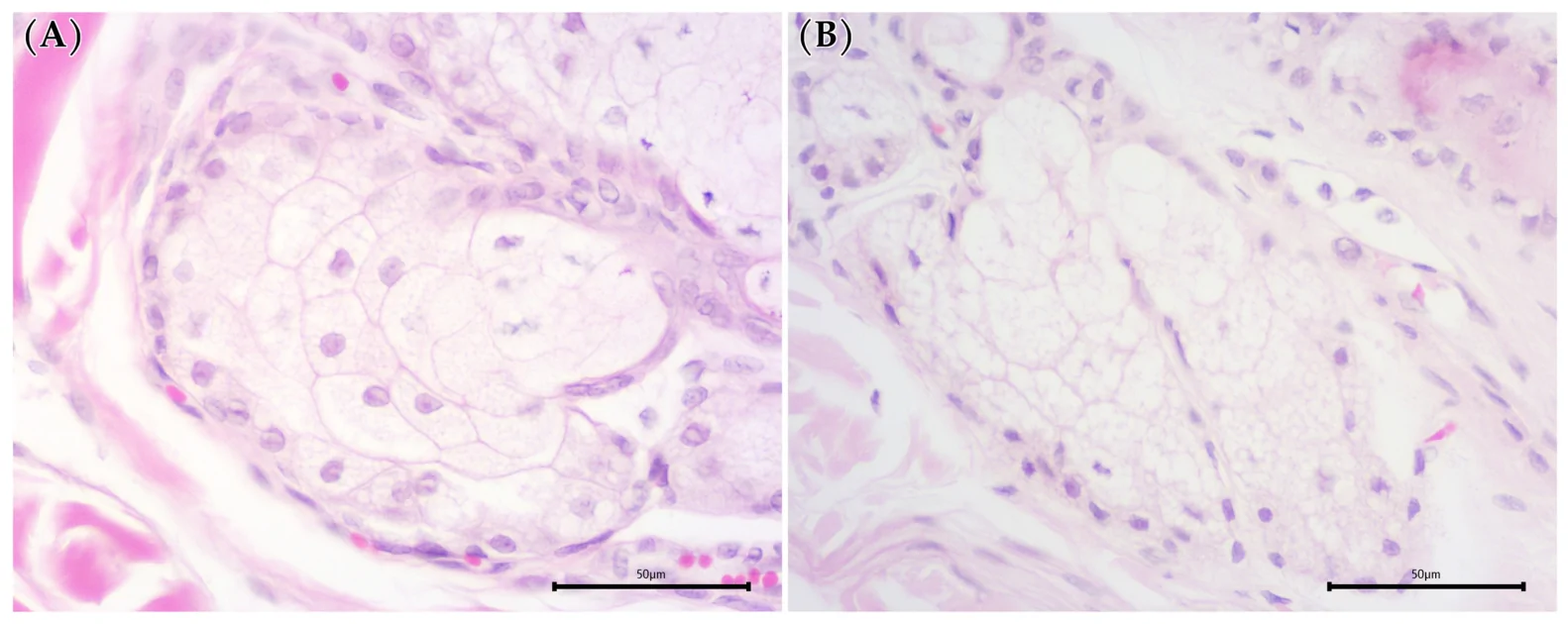
Postmortem autolytic stages of the sebaceous glands (H&E). (**A**) Normal architecture with the typical foamy cytoplasm of the sebocytes at 0 h (Stage 0), thigh left, scale bar = 50 µm; (**B**) vacuolization and nuclear pyknosis in the peripheral sebocytes at 72 h (Stage 1), thorax right, scale bar = 50 µm.

**Figure 6:**
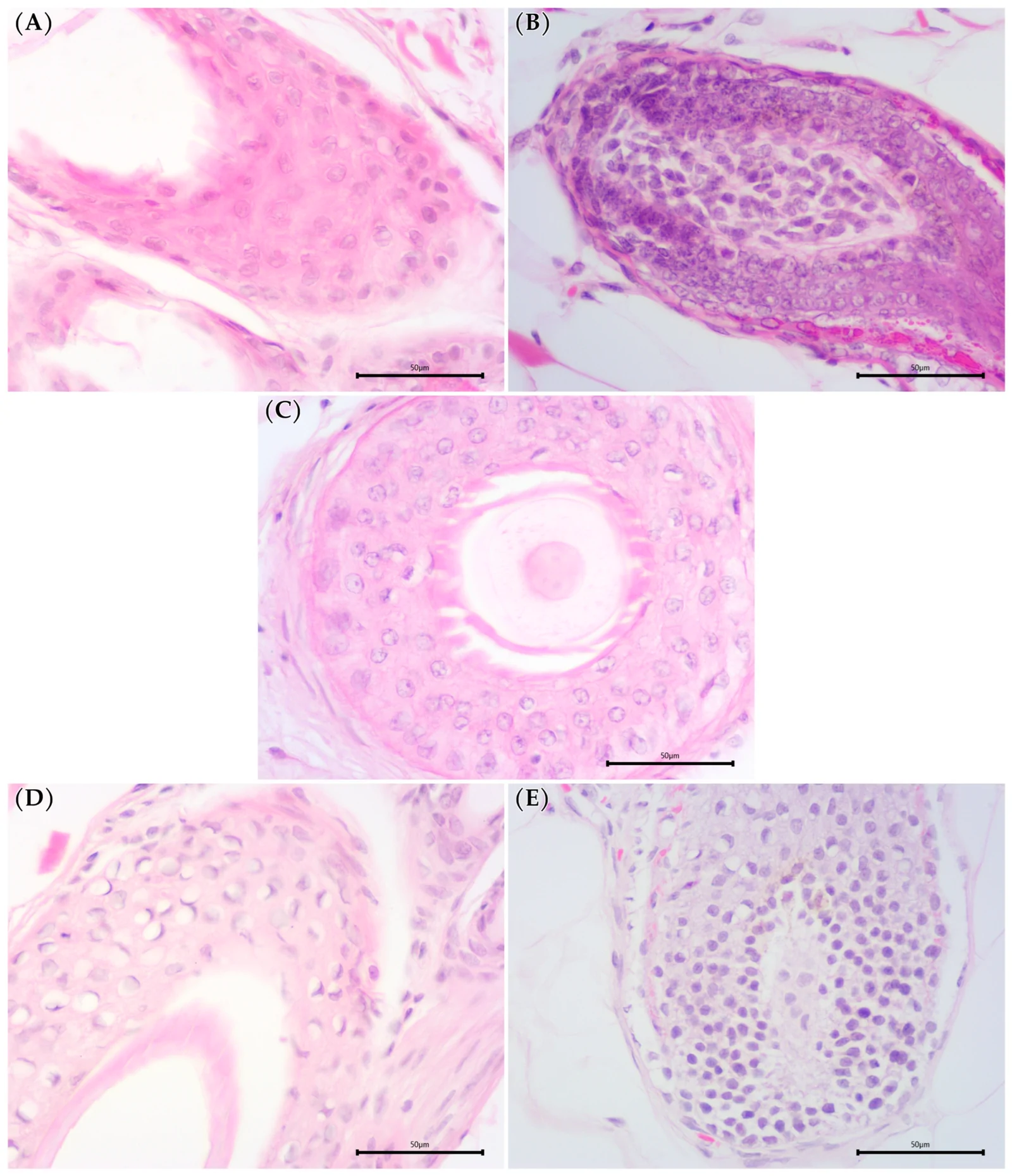
Postmortem autolytic stages of the hair follicles (H&E). (**A**) Isthmus with normal histology at 4 h (Stage 0), neck right, scale bar = 50 µm; (**B**) hair bulb with normal architecture at 8 h (Stage 0), abdomen right, scale bar = 50 µm; (**C**) isthmus showing vacuoles in sporadic outer root sheath cells at 24 h (Stage 1), thigh right, scale bar = 50 µm; (**D**) isthmus showing vacuolization of most outer root sheath cells at 72 h (Stage 2), neck right, scale bar = 50 µm; (**E**) hair bulb showing sporadic nuclear condensation at 144 h (Stage 2), neck left, scale bar = 50 µm.

**Table 1:** Individual characteristics of the nine dogs.

Dog	Age ^1^	Weight (kg)	BCS (/9)	Diagnosis (Cause of Euthanasia)	Month of Death
1	5–7	10	5/9	Spinal cord injury	December
2	8–10	9	4/9	Megaesophagus	December
3	5–7	12	5/9	Traumatic brain injury with coma	December
4	8–10	11	5/9	Non-reconstructable mandibular fracture	January
5	5–7	10	5/9	Spinal cord injury	January
6	8–10	9	4/9	Oral neoplasia ^2^	January
7	5–7	10	5/9	Spinal cord injury	January
8	5–7	10	5/9	Idiopathic epilepsy	February
9	8–10	11	5/9	Tracheal collapse	February

^1^ Age estimated from dentition. ^2^ Locally advanced and non-resectable; not histologically typed.

**Table 2:** Histomorphological criteria for postmortem staging of the sweat glands.

Sweat Glands	Histological Features
Stage 0	Normal architecture with intact epithelium
Stage 1	– Cytoplasmic vacuolization of the epithelial cells – Sporadic nuclear pyknosis – Widening of the interstitial gaps between cells
Stage 2	– Loss of glandular architecture – Detachment and collapse of the secretory epithelium into the lumen – Nuclear pyknosis or karyolysis in most epithelial cells – Cytoplasmic vacuolization of cells still attached to the basement membrane (not shed into the lumen) – Persisting interstitial gaps between the remaining attached cells

**Table 3:** Histomorphological criteria for postmortem staging of the sebaceous glands.

Sebaceous Glands	Histological Features
Stage 0	Normal architecture with the typical foamy cytoplasm of the sebocytes
Stage 1	– Cytoplasmic vacuoles in the sebocytes, more sharply defined than the normal foamy appearance – Nuclear pyknosis or karyolysis in peripheral sebocytes

**Table 4:** Histomorphological criteria for postmortem staging of the hair follicles.

Hair Follicles	Histological Features
Stage 0	Normal architecture with intact root sheaths
Stage 1	– Vacuoles in sporadic outer root sheath cells at the isthmus – Nuclear pyknosis in sporadic outer root sheath cells at the isthmus – Bulb cells retaining normal morphology
Stage 2	– Vacuoles in most of the outer root sheath cells at the isthmus – Nuclear pyknosis or karyolysis in most of the outer root sheath cells at the isthmus – Sporadic nuclear pyknosis in the hair bulb cells, where present

**Table 5:** Distribution of sweat gland histomorphological stages by region, side and postmortem interval.

PMI	Neck R	Neck L	Thorax R	Thorax L	Abdomen R	Abdomen L	Thigh R	Thigh L
**0 h**	Stage 0	Stage 0	Stage 0	Stage 0	Stage 0	Stage 0	Stage 0	Stage 0
**4 h**	Stage 1	Stage 1	Stage 1	Stage 1	Stage 1	Stage 1	Stage 1	Stage 1
**8 h**	Stage 1	Stage 1	Stage 1	Stage 1	Stage 1	Stage 1	Stage 1	Stage 1
**12 h**	Stage 1	Stage 1	Stage 1	Stage 1	Stage 1	Stage 2	Stage 1	Stage 1
**24 h**	Stage 1	Stage 1	Stage 1	Stage 2	Stage 2	Stage 2	Stage 1	Stage 1
**48 h**	Stage 2	Stage 2	Stage 2	Stage 2	Stage 2	Stage 2	Stage 2	Stage 2
**72 h**	Stage 2	Stage 2	Stage 2	Stage 2	Stage 2	Stage 2	Stage 2	Stage 2
**96 h**	Stage 2	Stage 2	Stage 2	Stage 2	Stage 2	Stage 2	Stage 2	Stage 2
**120 h**	Stage 2	Stage 2	Stage 2	Stage 2	Stage 2	Stage 2	Stage 2	Stage 2
**144 h**	Stage 2	Stage 2	Stage 2	Stage 2	Stage 2	Stage 2	Stage 2	Stage 2
**168 h**	Stage 2	Stage 2	Stage 2	Stage 2	Stage 2	Stage 2	Stage 2	Stage 2

Note: Values are consensus stages per site and postmortem interval across the nine animals.

**Table 6:** Glycoprotein positivity in sweat gland secretory cells. (+) positive; (−) negative.

PMI	Secretory Cells	Basement Membrane
**0 h**	+	+
**4 h**	+	+
**8 h**	+	+
**12 h**	−	+
**24 h**	−	+
**48 h**	−	+
**72 h**	−	+
**96 h**	−	+
**120 h**	−	+
**144 h**	−	+
**168 h**	−	+

**Table 7:** Distribution of sebaceous gland histomorphological stages by region, side, and postmortem interval.

PMI	Neck R	Neck L	Thorax R	Thorax L	Abdomen R	Abdomen L	Thigh R	Thigh L
**0 h**	Stage 0	Stage 0	Stage 0	Stage 0	Stage 0	Stage 0	Stage 0	Stage 0
**4 h**	Stage 0	Stage 0	Stage 0	Stage 0	Stage 0	Stage 0	Stage 0	Stage 0
**8 h**	Stage 0	Stage 0	Stage 0	Stage 0	Stage 0	Stage 0	Stage 0	Stage 0
**12 h**	Stage 0	Stage 0	Stage 0	Stage 0	Stage 0	Stage 0	Stage 0	Stage 0
**24 h**	Stage 0	Stage 0	Stage 0	Stage 0	Stage 0	Stage 1	Stage 0	Stage 0
**48 h**	Stage 0	Stage 0	Stage 0	Stage 1	Stage 1	Stage 1	Stage 0	Stage 0
**72 h**	Stage 1	Stage 1	Stage 1	Stage 1	Stage 1	Stage 1	Stage 1	Stage 1
**96 h**	Stage 1	Stage 1	Stage 1	Stage 1	Stage 1	Stage 1	Stage 1	Stage 1
**120 h**	Stage 1	Stage 1	Stage 1	Stage 1	Stage 1	Stage 1	Stage 1	Stage 1
**144 h**	Stage 1	Stage 1	Stage 1	Stage 1	Stage 1	Stage 1	Stage 1	Stage 1
**168 h**	Stage 1	Stage 1	Stage 1	Stage 1	Stage 1	Stage 1	Stage 1	Stage 1

Note: Values are consensus stages per site and postmortem interval across the nine animals.

**Table 8:** Distribution of hair follicle histomorphological stages by region, side, and postmortem interval.

PMI	Neck R	Neck L	Thorax R	Thorax L	Abdomen R	Abdomen L	Thigh R	Thigh L
**0 h**	Stage 0	Stage 0	Stage 0	Stage 0	Stage 0	Stage 0	Stage 0	Stage 0
**4 h**	Stage 0	Stage 0	Stage 0	Stage 0	Stage 0	Stage 1	Stage 0	Stage 0
**8 h**	Stage 0	Stage 0	Stage 0	Stage 0	Stage 0	Stage 1	Stage 0	Stage 0
**12 h**	Stage 0	Stage 0	Stage 0	Stage 1	Stage 0	Stage 1	Stage 0	Stage 0
**24 h**	Stage 1	Stage 1	Stage 1	Stage 1	Stage 1	Stage 2	Stage 1	Stage 1
**48 h**	Stage 2	Stage 2	Stage 2	Stage 2	Stage 2	Stage 2	Stage 2	Stage 2
**72 h**	Stage 2	Stage 2	Stage 2	Stage 2	Stage 2	Stage 2	Stage 2	Stage 2
**96 h**	Stage 2	Stage 2	Stage 2	Stage 2	Stage 2	Stage 2	Stage 2	Stage 2
**120 h**	Stage 2	Stage 2	Stage 2	Stage 2	Stage 2	Stage 2	Stage 2	Stage 2
**144 h**	Stage 2	Stage 2	Stage 2	Stage 2	Stage 2	Stage 2	Stage 2	Stage 2
**168 h**	Stage 2	Stage 2	Stage 2	Stage 2	Stage 2	Stage 2	Stage 2	Stage 2

Note: Values are consensus stages per site and postmortem interval across the nine animals.

## Data Availability

The data supporting the findings of this study are contained within the article.
